# Scorpions and Scorpionism in Roudan County, Southern Iran

**Published:** 2019-12-31

**Authors:** Mehran Shahi, Reza Habibi-Masour, Mehrdad Salehi, Mehdi Ghasemi-Nang, Emadaddin Rafizad, Madineh Abbasi, Ahmad Ali Hanafi-Bojd

**Affiliations:** 1 Department of Medical Entomology and Vector Control, School of Public Health and Infectious and Tropical Diseases Research Center, Hormozgan Heath Institute, Hormozgan University of Medical Sciences, Bandar Abbas, Iran; 2 Department of Diseases Control, Hormozgan University of Medical Sciences, Roudan, Iran; 3 Department of Diseases Control, Hormozgan University of Medical Sciences, Bandar Abbas, Iran; 4 Department of Diseases Control, Hormozgan University of Medical Sciences, Bandar Abbas, Iran; 5 Department of Medical Entomology and Vector Control, School of Public Health, Tehran University of Medical Sciences, Tehran, Iran

**Keywords:** Distribution, Scorpion, Scorpion sting, Scorpionism, Iran

## Abstract

**Background:**

Every year, thousands of cases and many deaths from scorpion sting are reported in tropical areas of South and Southwestern parts of Iran. The aim of this study was to identify the fauna and dangerous species of scorpions in Roudan County, southern Iran.

**Methods:**

This descriptive study was conducted in 10 stations in Hormozgan Province. Scorpion sampling was done randomly by searching for their shelter and digging out their nests during day, and with the use of UV light during night from February 2013 to October 2014. Data of scorpion stings were obtained from health center of Hormozgan Province during 2014–2016.

**Results:**

Overall, 155 scorpions were collected on a set of eight species belonging to Buthidae and Hemiscorpiidae families. These species were identified as *Mesobuthus persicus*, *Mesobuthus phillipsi*, *Hottentotta schach*, *Odontobuthus doriae*, *Compsobuthus persicus*, *Orthochirus farzanpayi*, *Androctonus crassicauda* and *Hemiscorpius acanthocercus*. One thousand and twenty-seven cases of scorpion sting were recorded during 2014–2016 with a peak period in summer. Most of cases were <44yr old. Five out of six medically important scorpions in Iran were actively identified in the study area.

**Conclusion:**

Results of this study would greatly help to identify risk factors of scorpion sting in high-risk areas for planning, management and treatment of patients with scorpion sting in these areas.

## Introduction

Scorpion sting is one of the most important medical issues in tropical countries of the world, threatening the health of residents in these areas. Every year, thousand cases and many deaths from scorpion sting are reported in tropical areas of South and Southwestern parts of Iran.

Iranian scorpion fauna consists of 64 species with the highest frequency been related to Buthidae family (1). Among the known species of scorpions, the most reported deaths due to scorpionism are related to three species *Androctonus crassicauda*, *H. lepturus* and *H. acanthocercus* (2). More than 75% of deaths from scorpion sting occurred in the southern provinces of Iran including, Khuzestan, Hormozgan, Bushehr and Ilam each year (3). In the southern regions of Iran, the diversity and density of scorpions are very high. However, information on their fauna, ecology and distribution is very low (4).

Hormozgan Province is one of the most important foci of scorpion sting in southern part of Iran. Ea**c**h year, several cases of deaths are reported due to scorpion stings from this area (5, 6). Scientific research in the field of scorpions has been very limited in Hormozgan Province (7–9). By the end of year 2012, about six species of scorpions were identified in the Jask County located in eastern part of Hormozgan Province (10). In an earlier study conducted in Hormozgan Province, 20 scorpion species were reported from this area (11). A recent study at the provincial capital of Hormozgan Province reported 22 scorpion species in which 5 species belong to *Hemiscorpius* genus including, *H. acanthocercus*, *H. enischnochela*, *H. lepturus*, *H. persicus* and *H. gaillardia* (12). In southern region of Iran, *H. lepturus* is the major cause of death owing to scorpionism (13–18).

Roudan County is among the high-risk foci of scorpion sting in Hormozgan Province. Annually about 400 cases of scorpion sting are reported from this county. Two deaths due to scorpion stings have been reported in 2012 from this county. So far, no study has been done on the fauna of scorpions in Roudan County of Hormozgan Province. Due to the reported deaths from scorpion sting in this county, the present study was being conducted to determine the fauna and distribution of dangerous scorpions’ species in this area.

Therefore, the main objective of this study was to identify and the introduction of dangerous scorpions’ species to provide implement the best treatment method for patients in this high-risk area of southern part of Iran.

## Materials and Methods

### Study area

Roudan County is located in the eastern part of Hormozgan Province with land area of 3,725km^2^. This county is bordered to the southwestern part by Kerman Province. In terms of geographical features, this county is composed of two areas thus plains and mountains. Roudan County is located at latitude 27^°^ 27’ north and longitude 57^°^ 11’ east with an altitude of 150 to 700m above sea level. This county has sub-tropical climate with average annual rainfall of 250mm and mean relative humidity of about 45%. The average annual minimum and maximum temperature of this county ranges between 7 °C and 49 °C, respectively.

This descriptive cross-sectional study was conducted in 10 stations in Hormozgan Province (Table 1).

**Table 1.** Species of scorpions collected in Roudan County, southern Iran, 2014

**Collection site**	**Topography**	**Altitude (m)**	**Latitude and Longitude**	**Species**
**Faryiab**	Mountain	325	27° 28′ 10.96″ N, 57° 4′ 16.30″ E	*A. crassicauda*, *H. acanthocercus*, *M. phillipsi*, *M. persicus*, *Ho. schach*, *C. persicus*
**Rahdar**	Mountain	591	27° 36′ 58.97″ N, 57° 6′ 3.83″ E	*A. crassicauda*, *M. phillipsi*, *O. doriae*, *O. farzanpayi*
**Abnama**	Plain	218	27° 27′ 37.66″ N, 57° 15′ 21.14″ E	*A. crassicauda*, *M. phillipsi*, *M. persicus*
**Bika**	Plain	180	27° 21′ 17.60″ N, 57° 10′ 20.95″ E	*A. crassicauda*, *M. phillipsi*
**Berentin**	Plain	184.	27° 17′ 44.99″ N, 57° 14′ 59.51″ E	*A. crassicauda*, *M. phillipsi*
**Jaghin-e Shomali**	Plain	234	27° 13′ 28.48″ N, 57° 22′ 38.44″ E	*A. crassicauda*, *M. phillipsi*, *M. persicus*
**Jaghin-e Jonoobi**	Plain	212	27° 12′ 17.99″ N, 57° 20′ 41.33″ E	*A. crassicauda*, *M. phillipsi*, *M. persicus*, *O. doriae*
**Ziyarat Ali**	Mountain	456	27° 44′ 35.87″ N, 57° 13′ 57.55″ E	*H. acanthocercus*, *O. doriae*, *A. crassicauda*, *M. phillipsi*, *M. persicus*
**Roodkhane Bar**	Mountain	498	27° 49′ 45.64″ N, 57° 17′ 41.69″ E	*H. acanthocercus*, *O. doriae*, *M. phillipsi*, *M. persicus*
**Mosafer Abad**	Plain	534	27° 52′ 54.94″ N, 57° 11′ 51.87″ E	*A. crassicauda*, *M. phillipsi*

### Scorpion samplings

Scorpions were collected from 10 sites in urban and rural areas of mountainous and plain regions (Table 1). Scorpion sampling was done randomly using hand catch method by searching under stones and clod, rift racks, under the bark of trees and other shelters, also digging out their nests during the day, and with the use of UV light at night from Feb 2013 to Oct 2014. The captured samples were placed in 75% alcohol container with an identification label. GPS device receiver (Garmin International) was used for recording the geographical coordinates.

The scorpions were identified by scorpiologist using morphological keys (11, 19) under an Nikon XN model stereomicroscope at Medical Entomology Department lab, Bandar Abbas City. The scorpion samples were kept in Entomology Lab, School of Public Health, Hormozgan University of Medical Sciences (HUMS), Bandar Abbas, Iran.

### Scorpion sting data Collection

Data about cases of scorpion sting in this descriptive cross-sectional study include, age, gender, site of the sting, geographical location of the scorpion sting, monthly cases, sting time, time referred to the hospital and total serum used in the treatment of patients were obtained from health center of Hormozgan Province during 2014–2016. Data analysis was performed using descriptive statistics, and graphs were plotted using Excel software.

## Results

### Scorpion fauna

**Fig. 1.**
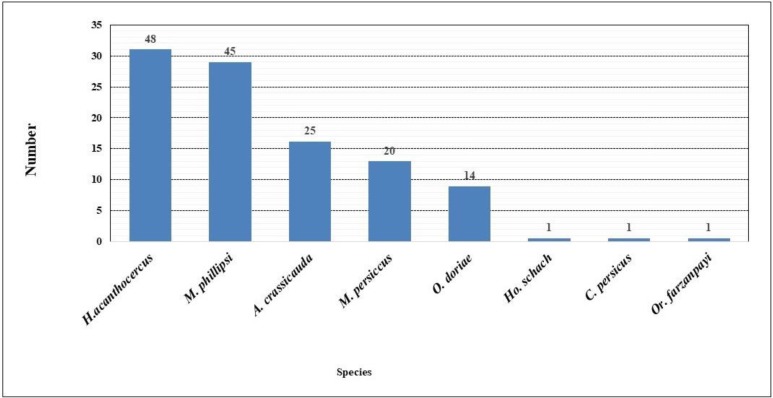
Species composition of scorpions collected in Roudan County, southern Iran, 2014

Overall, 155 scorpions were collected and identified in a set of 8 species, belonging to Buthidae and Hemiscorpiidae families. Of the total samples collected, 61 (39.3%) were female and 94 (60.7%) male. The family and habitat of the collected scorpion species were identified at each site of collection. *Androctonus crassicauda* and *Mesobuthus phillipsi* were collected from all stations in the study areas (Table 1). *Hemiscorpius acanthocercus* with a frequency collection of 48 was the predominant species. The lowest frequency was from *H. schach*, *C. persicus* and *O. farzanpayi* species (Fig. 1).

**Fig. 2.**
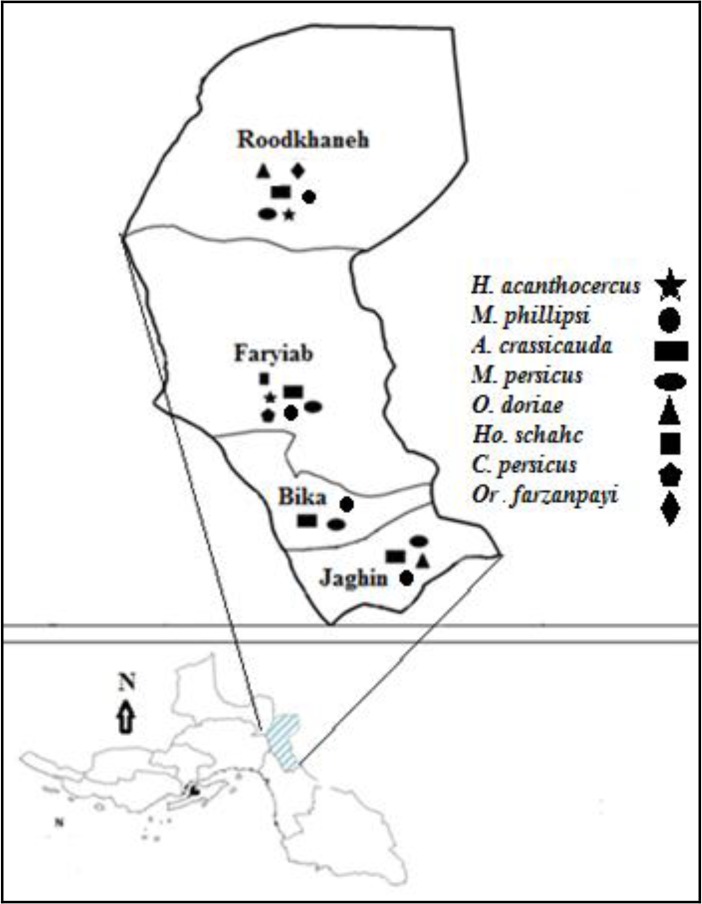
Distribution map of scorpion species in Roudan County, southern Iran, 2014

In this study, only one genus of the species *Hemiscorpius* was identified. This species was collected only from Roodkhaneh and Markazi Districts in mountainous area. The distribution map of the scorpion species identified has been shown in Fig. 2.

### Epidemiological data

**Fig. 3.**
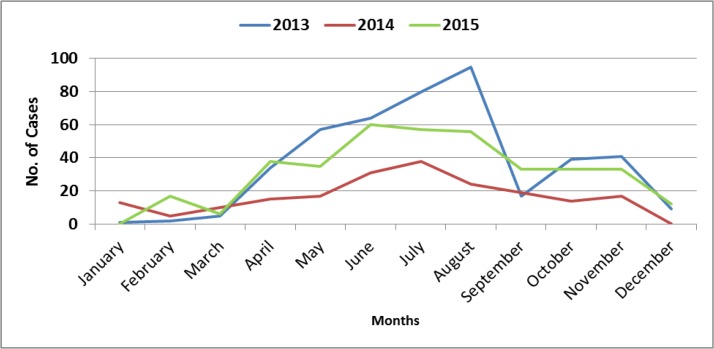
Distribution of cases of scorpion stings by months in Roudan County, southern Iran, 2014–2016

**Fig. 4.**
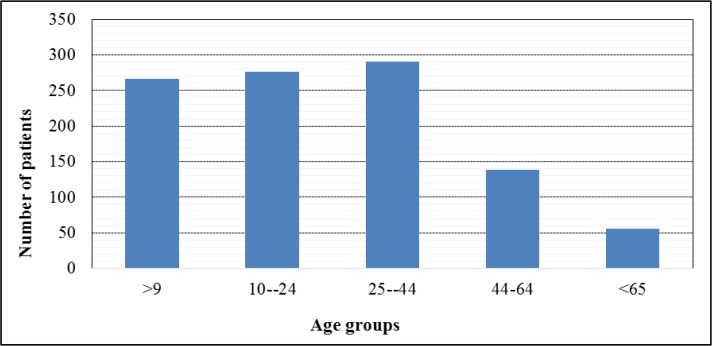
Patients age distribution of cases of scorpion sting in Roudan County, southern Iran, 2014–2016

The majority of scorpion stings in the study area happened in summer, with most cases (71.1%) been reported in rural areas (Fig. 3). According to the recorded evidence on scorpion sting checklist, in the rural and urban areas of Roudan the most cases of stinging were related to *Mesobuthus* genus scorpions. More people (36%) were stung in the first half of the night and the frequency of stings was higher among women (50.9%) than men. Aged 25 to 44yr (28.5%) were the most frequently affected age groups (Fig. 4). Most stings sites (41.2%) were in the hands of patients and more than half (54.6%) of patients were referred to treatment centers within one and half hours after the sting. Mean annual consumption of anti-scorpion venom was 510 vials. In general comparison, most cases of scorpion related to women occurred in the young and working age group in rural areas (Table 2).

**Table 2.** Cases of scorpion sting in Roudan County, southern Iran, 2012–2014

**Year**	**No. of cases**				**No. of antivenom doses**	**Age groups**				

	**Area**		**Gender**							
	
	**Urban**	**Rural**	**Male**	**Female**		**<9**	**10–24**	**25–44**	**45–64**	**>65**
**2014**	110	334	213	231	697	90	120	143	70	21
**2015**	46	157	106	97	257	62	55	49	21	16
**2016**	141	239	186	194	575	114	101	99	47	19
**Total**	297	730	505	522	1529	266	276	291	138	56

## Discussion

In this study, seven species of scorpions belonging to two families (Buthidae and Hemiscorpiidae) were identified in the study areas. Buthidae with seven species showed the highest frequency. Results of other studies in southern Iran about scorpion fauna were similar to our findings (7–10, 12). Most species, distribution and frequency of scorpions in our study were related to *M. phillipsi*, *M. persicus* and *A. crassicauda*. These three species were collected from all sampling sites; our findings in species composition were in accordance with other studies carried out in other counties of Hormozgan Province (10, 11). Results of studies in other parts of the country have also shown that *Mesobuthus* and *Androctonus* have the highest frequency (20–23). Distribution of *A. crassicauda* and *M. eupeus* has been reported from the northern and southern provinces in Iran (24–26). *Androctonus crassicauda* is one of the most dangerous scorpions’ species in Iran. Several cases of death from stings of this species have been reported from Hormozgan and Khuzestan provinces, in southern part of Iran (10, 27). Different species of this genus are adapted to different geographical conditions in Hormozgan Province.

The second family detected in the study area was Hemiscorpiidae. Only one species of this family named *H. acanthocercus* was identified. In this study, *H. acanthocercus* has been collected with high density in Roodkhaneh District. This district has so far, reported several cases of deaths from scorpion stings. However, Bandar Abbas County for the first time has reported one case of death from envenomation of *H. acanthocercus* venom (5). It seems this species is the main cause of this event in Roudan County. Previously, *H. acanthocercus* reported from Bandar Abbas and Khamir Counties in Hormozgan Province (11, 12, 19). Envenomation by this species cause severe complication or death (5). Lack of knowledge about this scorpion species among people and health care staff can be dangerous to health residents in their dispersal areas. *Hemiscorpius acanthocercus* is the main etiologic agent of death in Hormozgan Province (12). This scorpion genus is the most important cause of death from scorpion sting in Khuzestan Provinces (3, 16–18). According to the results of previous studies and the findings of the present study, *H. acanthocercus* has the highest distribution and the dominant species of the genus *Hemiscorpius* in mountainous areas of Hormozgan Province. The polyvalent anti-venom produced in Iran does not contain the venom of this scorpion.

In the present study, only one species of digger scorpion, *O. doriae* was collected. This species was reported from all parts of Iran (11, 24). *Odontobuthus species* distribution in most areas of Hormozgan Province has been reported in earlier studies (8, 10, 11). In this research, in daily scorpion catch, the *Odontobuthus* nests were mainly identified in the plain areas.

The lowest range distribution was related to *Or. farzanpayi* and *Ho*. *schach* These species were collected only from mountainous areas. Other researchers have reported similar results (11, 25, 28). *Hottentotta schach* and *Ho. saulcyi* have also been reported from mountainous area of Hormozgan Province including, Bandar Abbas, Khamir, Bastak, Parsian and Hajabad counties (8, 12). *Hottentotta schach* is one of the six medically important scorpions in Iran that has been reported widely distribution in all parts of country (24, 29). *Hottentotta jayakari* and *M. eupeus* were reported in previous studies from Abu-Moosa, Great Tunb, Hengam and Qeshm Islands in the Persian Gulf (7, 9).

This epidemiological study showed that the annual epidemiological factors of scorpion sting have a consistent pattern in Roudan County. Most cases of scorpion sting were in the summer. The results of other studies about scorpion sting in Iran are similar to our study results (30, 31). Scorpions have hibernation and because of the favorable temperatures in the warmer seasons, they are more active. This results in increased contact with residents and increased scorpion sting cases.

The highest percentage (71.1%) of sting occurred in rural areas and this is consistent with other researchers results in Iran (32). Similar results have also been reported in Turkey and Saudi Arabia (33–35). Sitting and sleeping on the ground, walking barefooted out of house, and putting clothing and bedding on the floor are the effective factors that increase scorpion sting in rural areas. Rural areas are ideal habitat for scorpions due to ecological and ecological conditions.

In terms of sting time, most stings occurred in the night. Scorpions are nocturnal arthropods and come out from their shelters after sunset. The scorpion’s nightly search for nourishment enters them into residential areas and increases the risk of scorpion sting. The frequency of scorpions’ sting in women was higher than men. This is in accordance with results of other researchers in Iran (36–39). Results of studies conducted in Saudi Arabia did not match our finding (40, 41). These differences in results might have occurred due to cultural differences between tribes in different areas. In high-risk areas, some activities such as collecting firewood and working in the agricultural fields raised the risk of a sting.

In our study, the highest percentage of scorpion stings was observed among 25–44yr old patients with the next high-risk groups being 10 to 24yr old group. These results were in agreement with some other studies (30, 42, 43). These two age groups have the most social activity in rural areas. The activities of these age groups in agricultural lands, gardens and desert areas put them at greater risk. The high rate of sting in the high-risk group of children also indicates the need to pay attention to the implementation of the scorpion prevention program in these areas. Comparing the results of this study showed that most cases of scorpions in rural areas occurred among women and youth age groups. It can be of great help to identify the risk factors of scorpion sting for planning, management and appropriate treatment of scorpion sting among patients in high-risk areas of South part of Iran.

## Conclusion

Due to high frequency of scorpion sting cases and the presence of *H. acanthocercus* in mountainous areas, hilly villages of Roudan County are located in high risk zone of scorpion sting in Hormozgan Province, southern parts of Iran. Therefore, planning should be focused on preventive measures including training of residents of these high-risk areas, health personnel and immediate on the treatment of patients with a scorpion sting in these areas.
